# Implementation gap in prehospital termination-of-resuscitation policies for out-of-hospital cardiac arrest with do-not-attempt-cardiopulmonary-resuscitation wishes in Japan: a nationwide survey

**DOI:** 10.1016/j.resplu.2026.101474

**Published:** 2026-09-01

**Authors:** Ryo Tanabe, Takashi Hongo, Tsuyoshi Nojima, Hiromichi Naito, Atsunori Nakao, Tetsuya Yumoto

**Affiliations:** a Department of Emergency, Critical Care, and Disaster Medicine, Faculty of Medicine, Dentistry, and Pharmaceutical Sciences, Okayama University, Okayama, Japan; b Department of Emergency, Critical Care, and Disaster Medicine, Kumamoto University, Kumamoto, Japan

**Keywords:** Prehospital care, Termination of resuscitation, Do-not-attempt-cardiopulmonary-resuscitation, Cardiac arrest, Medical control, Health policy implementation

## Abstract

**Aim:**

We evaluated the nationwide availability of prehospital termination-of-resuscitation (TOR) policies for out-of-hospital cardiac arrest (OHCA) with do-not-attempt-cardiopulmonary-resuscitation (DNACPR) wishes in Japan, and quantified the implementation gap between policy adoption and operational use.

**Methods:**

We conducted a nationwide cross-sectional survey of all prefecture-level and regional medical control councils and fire departments in Japan. The primary outcome was the fire-department-level crude operational application rate among departments with a policy in place.

**Results:**

Responses were obtained from 46 of 47 prefecture-level medical control councils (98%), 129 of 250 regional medical control councils (52%), and 561 of 791 fire departments (71%). A prehospital TOR policy for OHCA patients with DNACPR wishes was available in 265 of 561 fire departments (47%). Among 391 fire departments reporting at least one OHCA with DNACPR wishes in 2024, 156 had a policy in place. Among 151 with evaluable data, 78 (52%) did not apply the policy to any such case during the study year; 35 of these departments reported three or more DNACPR cases. Departments with a policy reported fewer transports with ongoing cardiopulmonary resuscitation than departments without a policy (46% vs. 82%, adjusted OR, 0.17; 95% CI, 0.09–0.30).

**Conclusion:**

Although prehospital TOR policies for OHCA patients with DNACPR wishes were available in nearly half of fire departments, implementation was highly heterogeneous: more than half of departments with a policy reported a crude operational application rate of 0% during the study year, despite encountering relevant cases. Efforts to improve operational uptake may be as important as policy development itself.

## Introduction

Out-of-hospital cardiac arrest (OHCA) may occur in patients who have previously expressed wishes to forgo resuscitation. However, emergency medical services (EMS) personnel may be unable to confirm or implement these wishes, leading to the initiation of resuscitation efforts despite the patient's preferences. Studies from several countries have shown that a substantial proportion of patients with OHCA have advance directives or do-not-attempt-cardiopulmonary-resuscitation (DNACPR) instructions in place. Nevertheless, resuscitation attempts and advanced life support interventions are frequently initiated in these patients in the prehospital setting.^1^, ^2^, ^3^, ^4^

These findings suggest that the gap between patients' DNACPR wishes and actual prehospital care occurs in many countries. However, the legal frameworks and operational procedures for honoring those wishes vary substantially across EMS systems. In Japan, EMS personnel often face difficulties when responding to OHCA patients with DNACPR wishes, and no nationally standardized prehospital protocol for withholding or terminating resuscitation has been established.^5^, ^6^

Whether the availability of a formal policy translates into its consistent application in practice remains unclear. For OHCA patients with DNACPR wishes, little is known about the nationwide availability of prehospital termination-of-resuscitation (TOR) policies in Japan, how consistently such policies are applied in practice, or whether prehospital case disposition differs according to policy availability. In this study, “termination of resuscitation” refers to withholding or discontinuing prehospital resuscitation after a patient’s previously expressed DNACPR wishes have been confirmed. We use the term “TOR policy” to describe policies governing these responses, which are conceptually distinct from conventional prognostic TOR rules based on arrest characteristics and response to treatment.

Importantly, translating policy availability into consistent field application may depend on multiple organizational and operational factors, including verification requirements, physician availability, legal clarity, EMS training, family acceptance, organizational culture, and case-level eligibility. These potential barriers and facilitators can be conceptualized using implementation-science frameworks such as the Consolidated Framework for Implementation Research, which may help distinguish policy availability from actual operational uptake.^7^

To address these knowledge gaps, we conducted a nationwide survey of prefecture-level and regional medical control (MC) organizations and fire departments responsible for field EMS operations in Japan. The objectives of this study were to assess the availability of prehospital TOR policies for OHCA patients with DNACPR wishes, characterize their operational uptake among fire departments with a policy in place, and examine whether prehospital case disposition differed according to policy availability. We hypothesized that policy availability would not necessarily translate into consistent operational uptake and that prehospital case disposition would differ according to policy availability.

## Methods

### Study design and setting

We conducted a nationwide cross-sectional survey study in Japan to evaluate the availability and operational use of prehospital TOR policies for OHCA with DNACPR wishes. The study focused on the implementation gap between policy availability and actual policy application.

In Japan, EMS is primarily operated by municipal fire departments. In the absence of a legal framework permitting prehospital TOR, EMS personnel are generally expected to initiate resuscitation and transport OHCA patients unless obvious biological signs of death are present.^5^ In 2017, the Japanese Society for Emergency Medicine issued a statement providing guidance on prehospital responses to patients in the final stage of life who wish to forgo resuscitation, offering a framework for local medical control organizations to develop region-specific protocols.^8^ In the absence of a unified national standard, prehospital TOR policies for OHCA with DNACPR wishes have been developed independently at the local medical control level.

The governance structure relevant to this study operates at three levels: prefecture-level MC councils, regional MC councils, and fire departments. Prefecture-level MC councils provide system-level oversight across an entire prefecture and may establish a prefecture-wide prehospital TOR policy; when such a policy is in place, regional MC councils within the prefecture operate under that framework. In prefectures without a prefecture-level policy, regional MC councils may independently develop and operate their own policies. Fire departments are the operational units that directly manage OHCA in the field.

Policy availability and policy characteristics were assessed in 2025, whereas fire-department-level case volume and prehospital outcomes were collected retrospectively for calendar year 2024. For analyses of 2024 fire-department-level case data, departments were classified according to policy status during 2024. Fire departments in which a policy had not yet been implemented at the time of the 2024 case accrual were classified as no policy for those analyses.

### Study population and survey framework

The survey targeted all 47 prefecture-level MC councils, 250 regional MC councils, and 791 fire departments in Japan, and was administered using an online survey platform (Google Forms). Two separate survey instruments were used. The policy survey, which assessed policy availability and characteristics, was first distributed to the EMS administrative officer at each of the 47 prefecture-level MC councils; respondents then forwarded the survey to the fire department serving as the administrative office of each regional MC council within their prefecture. A separate case-data survey was subsequently distributed to all 791 fire departments via the EMS administrative officer for each medical control area to collect retrospective aggregate data on OHCA cases with DNACPR wishes for calendar year 2024. Both surveys were non-anonymous, no reminders were sent, and both were conducted from November 25 to December 12, 2025. The MC survey addressed policy availability, policy characteristics (including verification procedures, education and training, and year of implementation), and governance-level information; the complete survey instrument is provided in Appendix A.

MC-level analyses described policy availability and policy characteristics separately for prefecture-level and regional MC councils, because these two organizational levels differ in geographic scope and governance authority. Fire-department-level comparative analyses included departments reporting at least one OHCA case with DNACPR wishes in 2024 and classified as having either a policy in place or no policy. The implementation-gap analysis included fire departments with a policy in place and at least one OHCA case with DNACPR wishes in 2024.

### MC survey and fire department survey

The MC survey evaluated whether each responding MC organization had a prehospital TOR policy for OHCA with DNACPR wishes and, when present, described its operational characteristics. The fire-department survey collected retrospective aggregate data for calendar year 2024. Fire departments reported total OHCA cases, OHCA cases with DNACPR wishes, selected case characteristics, and prehospital disposition outcomes. Variables included written documentation, arrest location, transport with cardiopulmonary resuscitation (CPR), on-scene termination under policy, and complaints or objections from family members (Appendix B).

### Definitions

For this survey, a prehospital TOR policy was defined as a procedure that allows EMS personnel to terminate resuscitation when the patient's DNACPR wishes can be confirmed, for example through written documentation or confirmation with the patient's primary physician or a hospital-based physician providing real-time remote medical direction to EMS personnel by telephone. OHCA with DNACPR wishes was defined according to respondent report. The survey instrument (Appendix B) defined DNACPR as “a previously expressed wish not to receive cardiopulmonary resuscitation in the event of cardiac arrest” and did not require a specific form of evidence (e.g., written documentation, verbal family report, physician confirmation, or an advance directive) for a case to be counted. Written documentation of DNACPR wishes was recorded separately as a distinct case characteristic, rather than as a requirement for case ascertainment. Policy application referred to the use of a policy in an OHCA case with DNACPR wishes during 2024. Crude operational application rate was defined for each fire department as policy-applied OHCA cases with DNACPR wishes divided by all OHCA cases with DNACPR wishes. Because the survey did not capture case-level eligibility for policy use, this measure reflects fire-department-level policy application, or operational uptake, rather than adherence among eligible cases. Transport with CPR was defined as transport to hospital with ongoing CPR. On-scene termination was defined as prehospital termination under a policy pathway and was assessed only in departments with a policy in place, because departments without a policy would generally continue resuscitation and transport the patient rather than terminate resuscitation in the field.

### Outcomes

The primary outcome was fire-department-level crude operational application rate in 2024 among departments with a policy in place. This outcome was used to quantify the implementation gap after policy adoption. Secondary outcomes were policy availability at the MC-organization and fire department levels, each defined as the proportion of responding organizations reporting a policy in place. Additional secondary outcomes were transport with CPR, on-scene termination under policy, and complaints or objections from family members.

### Statistical analysis

MC-level and fire department-level data were analyzed separately. Descriptive data are presented as number (percentage) or median with interquartile range (IQR) as appropriate. Analyses were conducted using available data for each variable; therefore, denominators may vary because of missing responses. Percentages were calculated using the number of non-missing responses as the denominator. The distribution of fire department-level crude operational application rates among departments with a policy in place was summarized using the median, IQR, and range, and was further stratified by annual DNACPR case volume (1–5, 6–15, 16–30, and ≥31 cases). To assess whether the concentration of very low application rates could be explained by denominator instability in low-volume departments, we additionally used an empirical-Bayes random-intercept logistic model to obtain shrinkage-adjusted estimates of department-level application rates. Because fire departments are nested within regional MC councils and OHCA cases within a fire department are unlikely to be independent, comparisons between fire departments with a policy in place and those with no policy in Table 2 were based on mixed-effects logistic regression models with a random intercept for regional MC council, reported as adjusted odds ratios (ORs) with 95% confidence intervals (CIs); fire departments with no regional MC council affiliation were treated as independent (singleton) clusters. For complaints or objections from family members, the model did not converge because of perfect separation (no complaints in the no-policy group), and a case-level Fisher's exact test was used instead.

To assess potential non-response bias among regional MC councils, we performed an additional analysis comparing regional-level survey response rates between councils located in prefectures with and without a prefecture-wide TOR protocol. Response rates were compared using Fisher's exact test.

As sensitivity analyses, we assessed the crude operational application rate after restricting the analysis to fire departments reporting ≥3 and ≥5 OHCA cases with DNACPR wishes in 2024. We also compared the crude operational application rate according to the verification pathway of the corresponding MC organization, categorized as less restrictive (documentation or physician confirmation alone was sufficient) or combination-required (both documentation and physician confirmation were required). Application rates between verification-pathway groups were compared using the Wilcoxon rank-sum test.

Statistical analyses were performed using Stata/SE 18.0.

### Ethics

This study was approved by the Okayama University Hospital Ethics Committee (K2511-031) and was conducted in accordance with the Declaration of Helsinki. Participation in both surveys was voluntary; respondents provided consent prior to completing the questionnaire.

## Results

### Survey response and nationwide policy availability

**Fig. 1:**
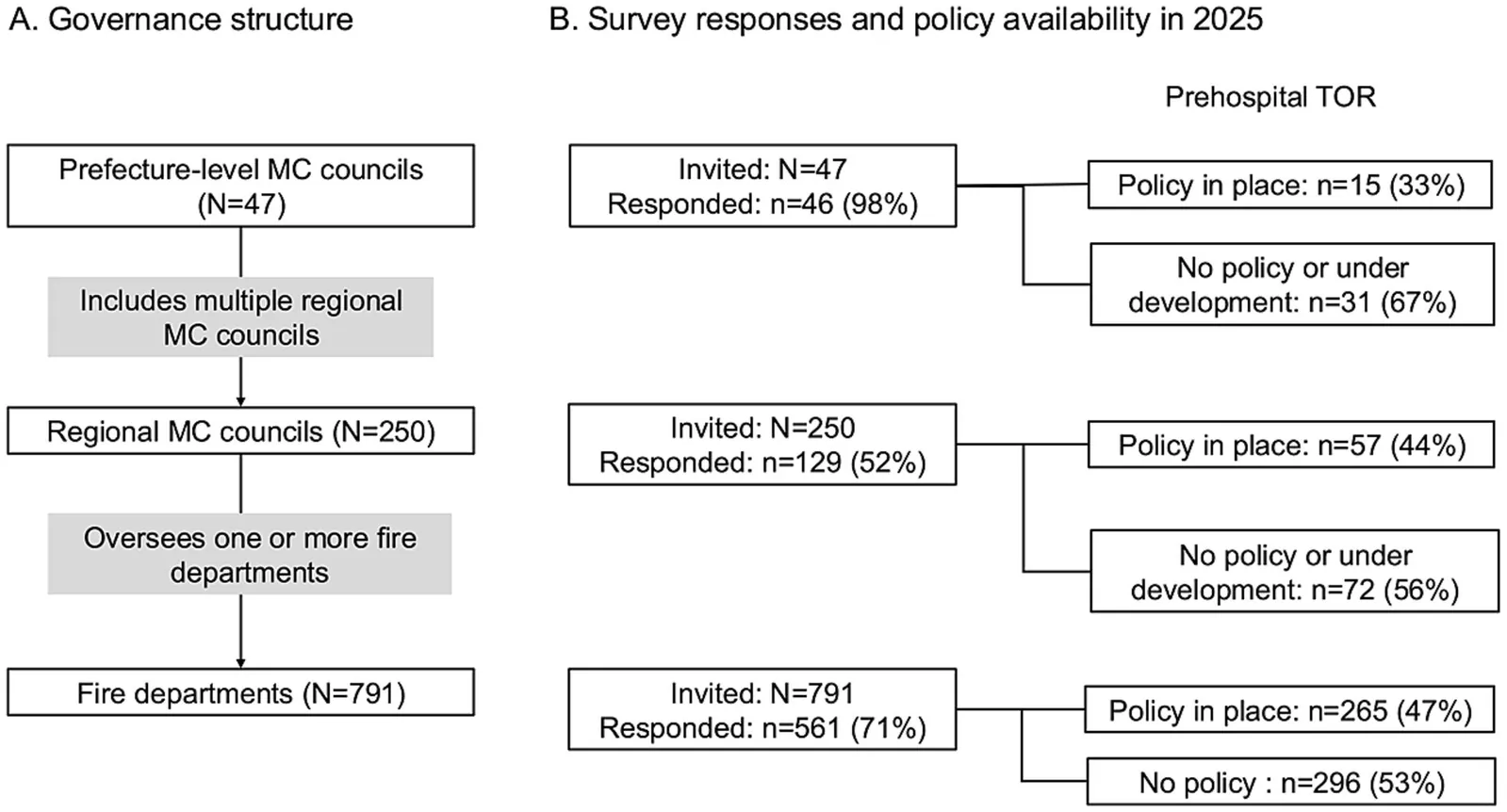
Governance structure, survey response, and policy availability for prehospital TOR in OHCA with DNACPR wishes in Japan.

The survey covered three organizational levels: prefecture-level MC councils, regional MC councils, and fire departments (Fig. 1). Response rates were 98% (46/47) for prefecture-level MC councils, 52% (129/250) for regional MC councils, and 71% (561/791) for fire departments. A prehospital TOR policy for OHCA with DNACPR wishes was reported to be in place in 265 of 561 responding fire departments (47%).

**Supplementary Fig. 1:**
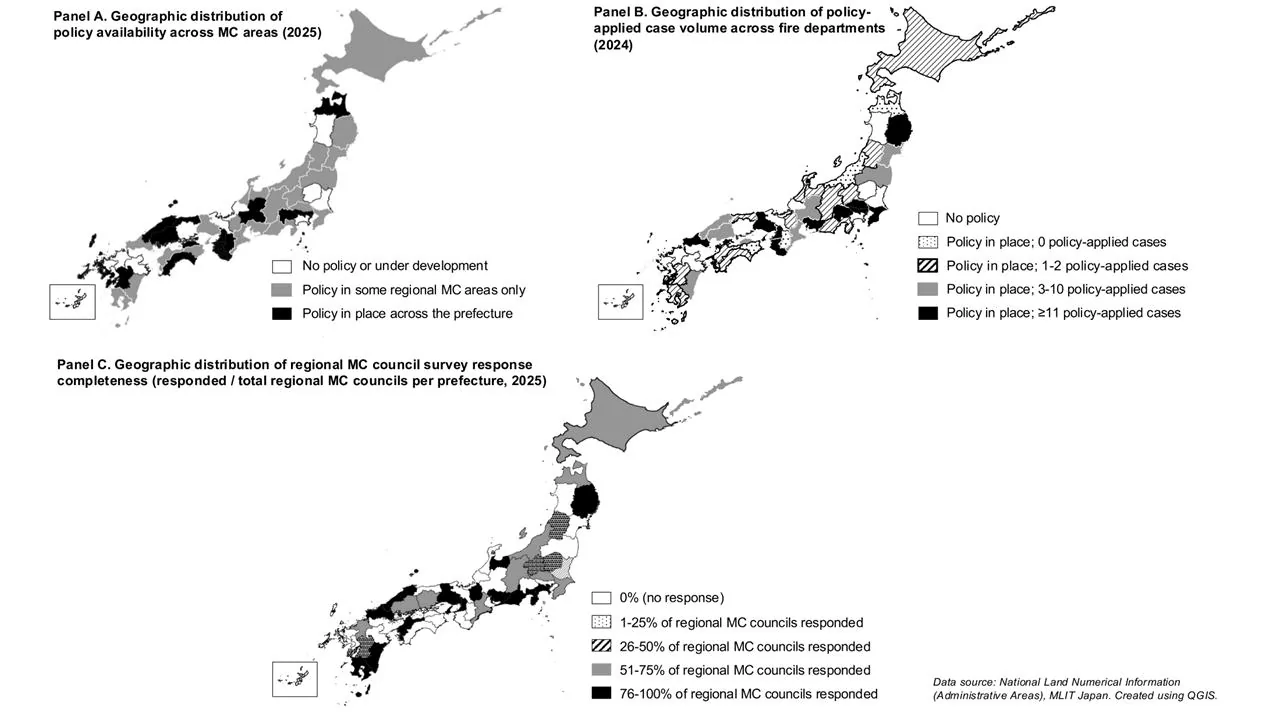
Geographic distribution of prehospital TOR policies by MC area in Japan (2025), policy-applied case volume by fire department (2024), and regional MC council survey response completeness by prefecture (2025).

Among 46 responding prefecture-level MC councils, 15 (33%) reported a policy in place, 8 (17%) reported a policy under development, and 23 (50%) reported no policy. Among 129 responding regional MC councils, 57 (44%) reported a policy in place, 22 (17%) reported a policy under development, and 50 (39%) reported no policy (Table 1). Regional MC councils located in prefectures with a prefecture-wide TOR protocol had a lower response rate than those located in prefectures without such a protocol (23 of 62 [37%] vs. 90 of 141 [64%]; *P* < 0.001). The geographic distribution of regional MC council response completeness by prefecture is shown in Supplementary Fig. 1C. Geographic variation in policy availability is shown in Supplementary Fig. 1A, in which each prefecture is color-coded according to whether a policy was in place across the prefecture, at only some regional MC areas, or not in place or under development.

**Table 1:** Policy status and characteristics of prehospital TOR policies for OHCA with DNACPR wishes across MC organizations in Japan.

**Policy status**	**Prefecture-level MC council (*N* = 46)**	**Regional MC council (*N* = 129)**
Policy in place	15 (33)	57 (44)
Under development	8 (17)	22 (17)
No policy	23 (50)	50 (39)

**Policy characteristics and implementation readiness**	**Prefecture-level MC council** **(*N* = 15)**	**Regional MC council** **(*N* = 57)**
**Maturity**		
Year of implementation, median (IQR)	2023 (2019–2025)	2021 (2019–2024) (*n* = 56)
**Policy revision status**		
No revision since implementation	12 (80)	36 (63)
Revised at least once	2 (13)	13 (23)
Periodically reviewed or updated	1 (7)	8 (14)

**Verification and authorization**		
**Confirmation of DNACPR wishes by EMS**		
Not routinely performed	9 (60)	37 (65)
Performed conditionally	3 (20)	15 (26)
Routinely performed	3 (20)	5 (9)
**Exclusion criteria for the TOR pathway, multiple responses**		
External causes (trauma/drowning/asphyxia, etc.)	12 (80)	43 (75)
Family strongly requests CPR	8 (53)	39 (68)
Pediatrics (<18 years)	3 (20)	9 (16)
None specified	2 (13)	12 (21)
**Verification sources permitting TOR, multiple responses**		
Written documentation	1 (7)	7 (12)
Real-time phone confirmation with the primary physician only	3 (20)	20 (35)
Real-time phone confirmation with an online MC physician only	0 (0)	7 (12)
Written documentation plus real-time phone confirmation with the primary physician	10 (67)	34 (60)
Written documentation plus real-time phone confirmation with an online MC physician	0 (0)	15 (26)
**Authorized decision-maker for TOR, multiple responses**		
Primary physician	15 (100)	56 (98)
Online MC physician	0 (0)	18 (32)
EMS team leader	0 (0)	2 (4)

**Documentation and disposition**		
**Acceptance of written documentation**		
Any written documentation accepted	3 (20)	18 (32)
Accepted with conditions	1 (7)	12 (21)
Only the locally designated form accepted	4 (27)	5 (9)
Not specified	7 (47)	21 (37)
**Post-TOR disposition, multiple responses**		
Handover to the primary physician	11 (73)	50 (88)
Handover to family	9 (60)	29 (51)
Transport to hospital without CPR	3 (20)	16 (28)
Handover to police	0 (0)	1 (2)
**Time limits and forms**		
Maximum on-scene waiting time for physician arrival specified	5 (33)	25 (44)
Maximum on-scene waiting time before handover to family specified	4 (27)	16 (28)
Family signature or form required	9 (60)	31 (54)

**Implementation readiness**		
**Regional collaboration for advance care planning**		
Strong	1 (7)	6 (11)
Partial	2 (13)	26 (46)
None	12 (80)	25 (44)
**Legal issues clarified**		
Clear	3 (20)	2 (4)
Mostly understood	6 (40)	31 (54)
Not discussed	6 (40)	20 (35)
Unclear or concerns remain	0 (0)	4 (7)
**Education and training for EMS personnel, multiple responses**		
Training at policy introduction	2 (13)	37 (65)
Regular training	0 (0)	15 (26)
Manual distribution or self-learning	5 (33)	29 (51)
No education or training implemented	4 (27)	6 (11)
Other	5 (33)	2 (4)

Data are presented as No. (%) unless otherwise indicated. Percentages in the Policy status section were calculated among all responding organizations (*N* = 46 for prefecture-level and *N* = 129 for regional MC councils). Percentages in the Policy characteristics and implementation readiness section were calculated among organizations reporting a policy in place (*N* = 15 for prefecture-level and *N* = 57 for regional MC councils), unless a nested denominator is shown. Multiple-response items were not mutually exclusive; percentages may exceed 100%. For regional MC councils, year of implementation was missing for 1 of 57 councils with a policy in place (*n* = 56 with data).

*Abbreviations:* TOR, termination of resuscitation; OHCA, out-of-hospital cardiac arrest; DNACPR, do-not-attempt-cardiopulmonary-resuscitation; MC, medical control; IQR, interquartile range; EMS, emergency medical services; CPR, cardiopulmonary resuscitation.

**Table 2:** Case profile and prehospital disposition of OHCA with DNACPR wishes according to fire-department-level policy availability in 2024.

**Characteristic**	**Total**	**Policy in place**	**No policy**	**Adjusted OR (95% CI)**
**Study population**				
Fire departments reporting at least 1 OHCA case with DNACPR wishes, n	391	156	235	

**Case profile**				
OHCA with DNACPR wishes/total OHCA	3442/83,647 (4.1)	990/38,948 (2.5)	2,452/44,699 (5.5)	0.97 (0.79–1.20)
Written documentation	562/2,714 (21)	248/947 (26)	314/1,767 (18)	1.38 (0.82–2.31)
Arrest at home	1,784/3,434 (52)	540/989 (55)	1,244/2,445 (51)	1.16 (0.90–1.51)
Arrest in a long-term care facility	1,493/3,382 (44)	438/987 (44)	1,055/2,395 (44)	0.89 (0.68–1.15)

**Prehospital disposition outcomes**				
On-scene termination		408/965 (42)		
Transport with CPR	2,352/3,308 (71)	456/984 (46)	1,896/2,324 (82)	0.17 (0.09–0.30)

**Family-related concern**				
Complaints or objections from family members	2/2,679 (0.1)	2/946 (0.2)	0/1,733 (0.0)	Not estimable

Fire departments were the unit of recruitment and grouping. Values are *n*/*N* (%) of pooled OHCA cases with DNACPR wishes unless otherwise indicated; percentages use the non-missing denominator for each row. Because cases are nested within fire departments and fire departments within regional MC councils, adjusted ORs (95% CI) from mixed-effects logistic regression (see Methods) are shown instead of *P* values, except for complaints (not estimable; Fisher's exact test, *P* = 0.125). On-scene termination was assessed only among departments with a policy in place. Departments with counts exceeding the applicable denominator (a logical inconsistency) were excluded from the corresponding row (2, 3, 4, 4, 6, and 1 departments for DNACPR prevalence, documentation, arrest at home, arrest in a long-term care facility, transport with CPR, and on-scene termination, respectively).

*Abbreviations:* OHCA, out-of-hospital cardiac arrest; DNACPR, do-not-attempt-cardiopulmonary-resuscitation; CPR, cardiopulmonary resuscitation. OR, odds ratio; CI, confidence interval; MC, medical control.

### Policy characteristics reported by medical control organizations

**Supplementary Fig. 2:**
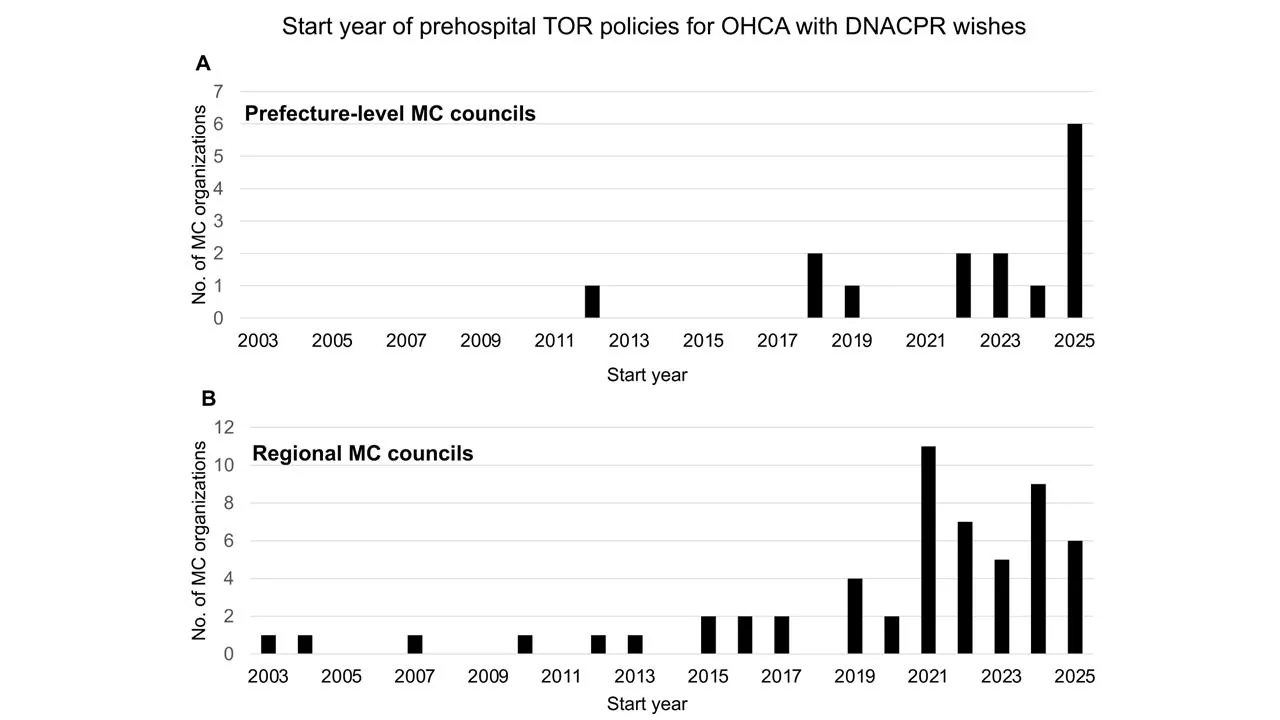
Distribution of implementation years for prehospital TOR policies by MC type.

Among the 15 prefecture-level MC councils reporting a policy in place, the median year of implementation was 2023 (IQR, 2019–2025); among 56 of 57 regional MC councils with a documented implementation year, the median year was 2021 (IQR, 2019–2024). The distribution of policy start years by MC type is shown in Supplementary Fig. 2 (Table 1). No revision since implementation was reported by 12 of 15 prefecture-level MC councils (80%) and 36 of 57 regional MC councils (63%).

For verification of DNACPR wishes, EMS confirmation was not routinely performed in 9 of 15 prefecture-level MC councils (60%) and 37 of 57 regional MC councils (65%); routine confirmation was reported by 3 (20%) and 5 (9%), respectively (Table 1). The most common verification pathway permitting termination was written documentation combined with real-time phone confirmation with the patient's primary physician, reported by 10 of 15 prefecture-level MC councils (67%) and 34 of 57 regional MC councils (60%). The patient's primary physician was the authorized decision-maker in all 15 prefecture-level MC councils (100%) and 56 of 57 regional MC councils (98%). Additional policy characteristics, including post-termination management and EMS training, are shown in Table 1.

### Case profile and prehospital disposition according to policy availability

**Supplementary Fig. 3:**
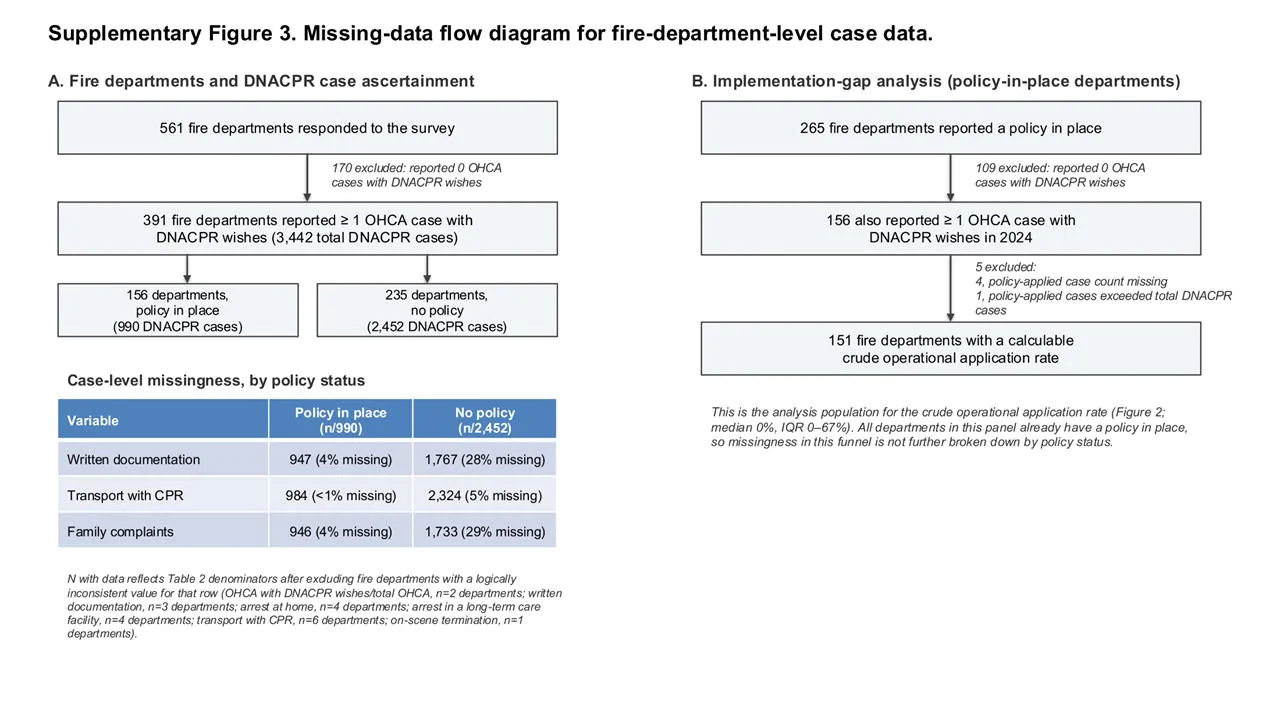
Missing-data flow diagram for fire-department-level case data.

A total of 391 fire departments reported at least one OHCA case with DNACPR wishes in 2024, including 156 with a policy in place and 235 with no policy (Table 2). These departments reported 83,647 total OHCA cases, of which 3442 (4.1%) involved DNACPR wishes—990 of 38,948 (2.5%) in departments with a policy in place and 2452 of 44,699 (5.5%) in those without. Although this pooled case-level difference appeared large, a mixed-effects logistic regression model accounting for clustering of fire departments within regional MC councils showed no significant association between policy availability and the proportion of OHCA cases with DNACPR wishes (adjusted OR, 0.97 [95% CI, 0.79–1.20]). Written documentation of DNACPR wishes was reported more often, at the pooled case-level, in departments with a policy in place than in those without (26% vs. 18%); however, this difference was not confirmed after accounting for clustering of fire departments within regional MC councils (adjusted OR, 1.38 [95% CI, 0.82–2.31]). Because responses were incomplete for some case-level items, analyses of prehospital disposition and family complaints were based on cases with available data. The number of fire departments and cases excluded at each step, and missingness by policy status, are shown in Supplementary Fig. 3.

Among OHCA cases with DNACPR wishes with available data, transport with CPR occurred in 2352 of 3308 cases (71%) overall, including 456 of 984 (46%) in departments with a policy in place and 1896 of 2324 (82%) in those without. This difference remained significant after accounting for clustering of fire departments within regional MC councils (adjusted OR, 0.17 [95% CI, 0.09–0.30]). In departments with a policy in place, on-scene termination under policy was reported in 408 of 965 OHCA cases with DNACPR wishes (42%) with available policy-application data (Table 2). Among OHCA cases with DNACPR wishes with available family-complaint data, complaints or objections from family members were rare, occurring in 2 of 2679 cases (0.1%), both in departments with a policy in place (2 of 946 [0.2%] vs. 0 of 1733 [0.0%]; *P* = 0.125).

### Implementation gap among fire departments with a policy in place

**Fig. 2:**
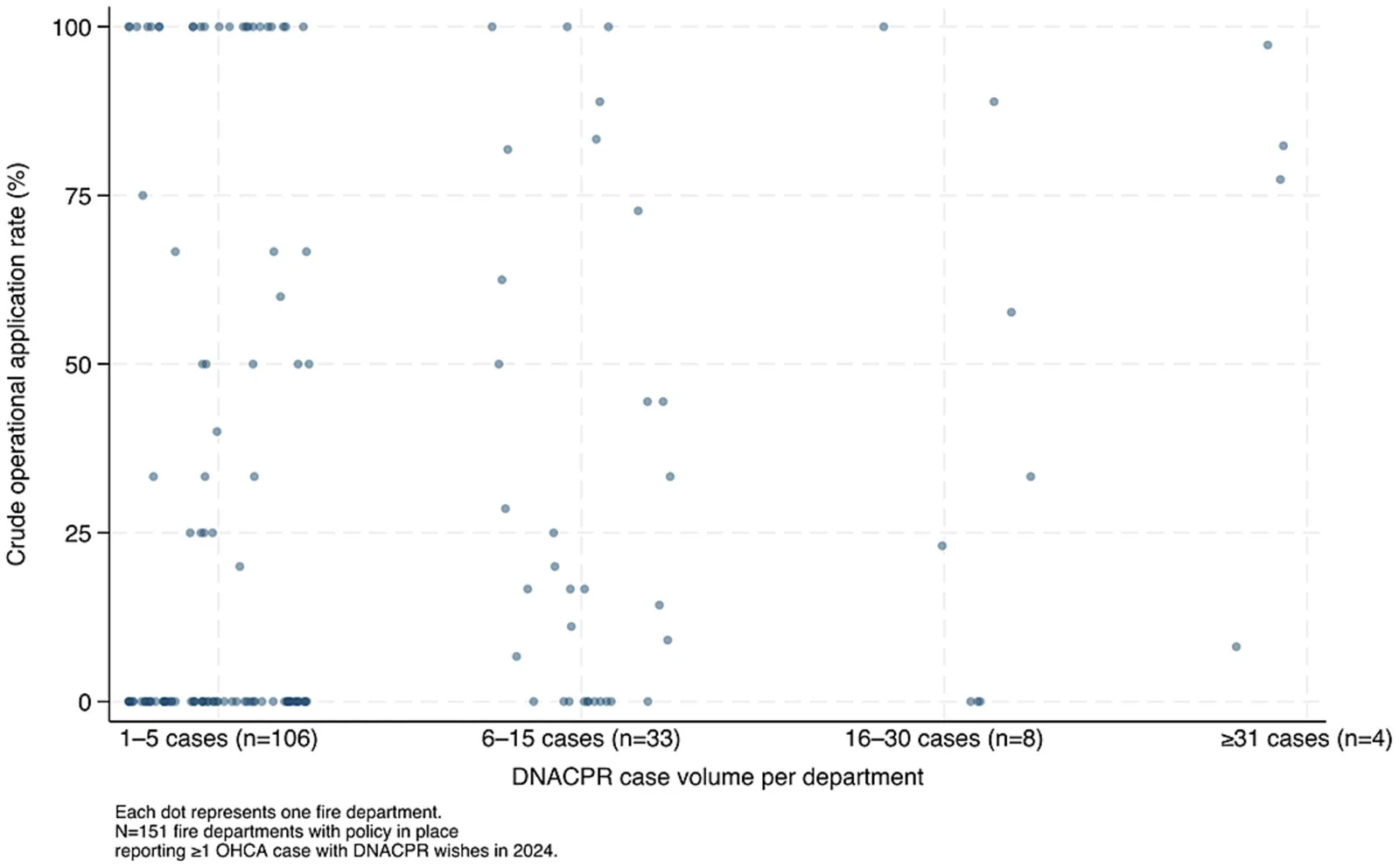
Crude operational application rates by annual DNACPR case volume among fire departments with a prehospital TOR policy in place in 2024.

Of 265 fire departments reporting a policy in place, 156 also reported at least one OHCA case with DNACPR wishes in 2024. Five departments were excluded from the implementation-gap analysis—4 because policy-applied case counts were missing and 1 because reported policy-applied cases exceeded the total number of DNACPR cases—leaving 151 departments with calculable crude operational application rates. Among these, 78 (52%) had a crude operational application rate of 0% (Fig. 2). Geographic variation in policy-applied case volume across fire departments is shown in Supplementary Fig. 1B. Among the 78 departments with a crude operational application rate of 0%, annual DNACPR case volume was 1 in 29 departments, 2 in 14, 3–4 in 19, 5–9 in 9, and ≥10 in 7; 35 of these 78 departments (45%) reported 3 or more DNACPR cases in 2024 despite not applying the policy. The median crude operational application rate across all 151 departments was 0% (IQR, 0–67%; range, 0–100%), and 27 of 151 departments (18%) had a 100% application rate. Stratified by DNACPR case volume, the median (IQR) and proportion of departments with a 0% application rate were 0% (0–67%; 60%) among departments with 1–5 cases, 17% (0–50%; 33%) among those with 6–15 cases, 28% (0–73%; 38%) among those with 16–30 cases, and 80% (43–90%; 0%) among those with ≥31 cases (Table 3). In an empirical-Bayes (random-intercept) logistic model, all 78 departments with a crude operational application rate of 0% had a shrinkage-adjusted probability of application below 5%, suggesting that the concentration at 0% was not explained solely by denominator instability in low-volume departments (Table 3). In a sensitivity analysis restricted to departments with ≥3 DNACPR cases (*n* = 88), 35 (40%) still had a 0% application rate, and among departments with ≥5 cases (*n* = 50), 16 (32%) had a 0% application rate. In an additional exploratory analysis of fire departments that could be linked to the verification pathway of the corresponding MC organization (*n* = 51), the mean crude operational application rate was numerically higher among departments under less restrictive verification pathways than among those under combination-required pathways (45% vs. 24%; *P* = 0.073) (Supplementary Table 2).

**Table 3:** Crude operational application rate by annual DNACPR case volume among fire departments with a policy in place in 2024.

**Annual DNACPR case volume**	***N***	**Median application rate, % (IQR)**	**0% application rate, *n* (%)**	**100% application rate, *n* (%)**	**Shrinkage-adjusted probability <5%, *n* (%)**
1–5	106	0 (0–67)	64 (60)	23 (22)	64 (60)
6–15	33	17 (0–50)	11 (33)	3 (9)	11 (33)
16–30	8	28 (0–73)	3 (38)	1 (13)	3 (38)
≥31	4	80 (43–90)	0 (0)	0 (0)	0 (0)

Total	151	0 (0–67)	78 (52)	27 (18)	78 (52)

Same population as Fig. 2 (*N* = 151 fire departments with a policy in place; see Fig. 2 legend for exclusion criteria). The shrinkage-adjusted probability was derived from an empirical-Bayes (random-intercept) logistic model partially pooling each department's estimate toward the overall mean; the same departments with a raw rate of exactly 0% also had a shrinkage-adjusted probability <5% in every stratum, indicating genuine non-application rather than denominator instability.

*Abbreviations:* DNACPR, do-not-attempt-cardiopulmonary-resuscitation; OHCA, out-of-hospital cardiac arrest; IQR, interquartile range; TOR, termination-of-resuscitation.

## Discussion

The key finding of this nationwide survey was a substantial implementation gap after policy adoption. Although prehospital TOR policies for OHCA with DNACPR wishes were available in nearly half of responding fire departments, implementation was highly heterogeneous: more than half of fire departments with a policy in place reported a crude operational application rate of 0%, and many departments did not apply the policy despite encountering relevant cases. Fire departments with a policy in place also reported less transport with CPR than those without a policy.

This interpretation is consistent with prior literature showing that advance directives or DNACPR orders do not reliably translate into concordant prehospital care and that EMS responses remain difficult when written and verbal DNACPR wishes coexist.^2^, ^3^, ^5^ Our findings add that policy availability alone did not ensure policy application.

The association between policy availability and case disposition should be interpreted cautiously. Departments with a policy in place may differ from those without in several important respects, including organizational maturity, physician oversight, regional practice patterns, resource availability, and local culture. These factors may independently influence clinical practice and represent potential sources of residual confounding that could partially explain the observed differences. Therefore, the observed association between policy availability and transport practices should not be interpreted as causal. Our data do not show that policy availability itself caused these differences.

The international context further supports the importance of distinguishing policy availability from operational use. As summarized in Supplementary Table 1, several countries and regions, including the United States, the United Kingdom, France, Germany, Japan, and Australia, have legal, professional, or protocol-based frameworks relevant to DNACPR wishes or prehospital TOR, but the basis for EMS authority, required confirmation, and decision-making pathways differs across systems.^3^, ^8^, ^9^, ^10^, ^11^, ^12^, ^13^, ^14^ In Japan, by contrast, no unified national DNACPR statute governs prehospital TOR; guidance from the Japanese Society for Emergency Medicine has provided a framework for local policy development, and local medical control policies in some regions provide the main operational basis.^8^ This comparison suggests that the presence of a legal or professional framework should not be equated with actual field-level application. Our results extend this point by showing that, even within a system where policies were increasingly available, policy application and crude operational application rates remained uneven.

Prior systematic evidence has raised concerns about both the evidence base for TOR rule implementation and the consistency of their application in practice.^15^, ^16^, ^17^, ^18^ Consistent with this pattern, a single-center study from Japan found that following the introduction of a formal prehospital TOR protocol in one city, the protocol was applied in approximately 60% of DNACPR cases, and a subset of cases still received advanced airway interventions despite the policy being in place.^13^ Our nationwide survey extends these observations: more than half of fire departments that had adopted a policy reported a crude operational application rate of 0%, and among these departments, 35 of 78 (45%) had reported 3 or more DNACPR cases in 2024, suggesting that low case volume alone does not account for non-application. Beyond case volume, several implementation domains may explain why policy availability did not consistently translate into application, including organizational readiness, variability in verification pathways, physician accessibility, EMS education and training, legal uncertainty, communication with families, or local governance structures.^19^ Although not all of these implementation determinants were directly measured in this survey, our MC-level data on verification pathways, decision authority, documentation requirements, legal clarity, and regional collaboration for advance care planning (Table 1) are broadly consistent with this implementation-science framework and may help explain why policy availability did not translate into consistent application. To examine this directly, we linked fire departments to their affiliated regional or prefecture-level MC council's policy characteristics using department-reported affiliation names; a name-matched MC record was found for 60% of fire departments in the implementation-gap analysis, but complete policy-characteristic data were available for 55%; physician authorization pathway used a separate crosswalk and was likewise available for 55%. In this matched, exploratory subset, none of the examined policy characteristics—including EMS training, documentation rules, physician authorization pathways, policy age, legal clarity, and regional collaboration—showed a strong or consistent association with the crude operational application rate. However, application rates were numerically higher among departments affiliated with MC organizations reporting clearer legal frameworks, stronger regional collaboration for advance care planning, or authorization extending to online MC physicians. A less restrictive verification pathway was also associated with a numerically higher mean application rate than a combination-required pathway (45% vs. 24%; Wilcoxon *P* = 0.073) (Supplementary Table 2). Given the incomplete linkage rate and small subgroup sizes, we consider these findings exploratory and hypothesis-generating rather than confirmatory.

This ambiguity may extend beyond the prehospital setting. When prehospital systems lack a workable pathway for verification and decision-making, the consequence may be not only continued transport with CPR but also ambiguity after hospital arrival. A Japanese survey reported frequent use of slow code—deliberately limited resuscitation rather than full resuscitative efforts—in OHCA patients with DNACPR orders after transport to hospital, suggesting that unresolved prehospital uncertainty may be carried into in-hospital care.^20^ Policy adoption alone may therefore be insufficient without a clear verification pathway, predefined decision authority, acceptable documentation standards, structured family and physician communication, and repeated EMS training.

### Limitations

This study has several limitations. First, its cross-sectional design precludes causal inference; differences in case disposition by policy availability should not be interpreted as direct policy effects. Differences in documentation, case recognition, arrest setting, physician access, regional practice patterns, and reporting completeness may explain part of this association. In particular, fire departments with and without TOR policies may have differed in how they identified and classified cases with DNACPR wishes. Accordingly, the observed difference in the reported proportion of OHCA cases with DNACPR wishes (2.5% vs. 5.5%) may reflect differential ascertainment rather than a true epidemiological difference. Fire department data were retrospective, aggregate, and self-reported, limiting patient-level adjustment, potentially introducing reporting error. The survey did not collect mutually exclusive and collectively exhaustive prehospital disposition categories; therefore, cases that were neither transported with ongoing CPR nor reported as terminated on scene under the policy could not be further classified. Transport data were missing for 5% of DNACPR cases (128 of 2452) in no-policy departments and less than 1% in policy departments. Second, because case-level eligibility for policy use was not captured, the crude operational application rate reflects operational uptake, not adherence among eligible cases. Consequently, the absence of policy application cannot necessarily be interpreted as implementation failure alone; other explanations, including lack of eligible cases, uncertainty regarding DNACPR verification, physician availability, family disagreement, or other operational factors, may also have contributed. Third, differential response rates and mixed organizational levels may have affected comparability. Only 52% of regional MC councils responded, raising the possibility of non-response bias. In an additional analysis, regional MC councils located in prefectures with a prefecture-wide TOR protocol were less likely to respond than those in prefectures without such a protocol, suggesting that the presence of an established prefecture-wide policy did not increase the likelihood of participation. However, among nonresponding regional MC councils in prefectures without a prefecture-wide TOR protocol, we could not determine whether locally established TOR protocols were present. Therefore, regional-level policy availability may have been incompletely ascertained, and residual non-response bias cannot be excluded. Accordingly, the reported policy availability should not be interpreted as a nationally representative estimate of TOR policy availability among all Japanese fire departments. Fourth, several differences that appeared significant using pooled case counts—including the proportion of OHCA with DNACPR wishes, written documentation, arrest at home, and arrest in a long-term care facility—were not confirmed after adjusting for clustering of fire departments within regional MC councils; only the difference in transport with CPR remained significant. These non-significant findings should not be interpreted as evidence of no true difference, given limited statistical power from the number and size of regional MC clusters. Prospective studies with case-level data are needed to evaluate the operational determinants of policy uptake.

## Conclusions

In this nationwide survey from Japan, prehospital TOR policies for OHCA with DNACPR wishes were available in nearly half of responding fire departments, but implementation was highly heterogeneous: more than half of fire departments with a policy in place reported a crude operational application rate of 0% despite encountering OHCA cases with DNACPR wishes, indicating a substantial implementation gap between policy adoption and operational use. Future efforts should focus not only on policy development but also on improving implementation in routine practice.

## Artificial intelligence use

OpenAI ChatGPT 5.4 and Anthropic Claude Sonnet 4.6 were used to support grammar and readability improvements and formatting. All outputs were critically reviewed and edited by the authors, who take full responsibility for the final manuscript.

## CRediT authorship contribution statement

**Ryo Tanabe:** Writing – review & editing, Writing – original draft, Methodology, Investigation, Funding acquisition, Formal analysis, Data curation, Conceptualization. **Takashi Hongo:** Writing – review & editing, Writing – original draft, Project administration, Methodology, Conceptualization. **Tsuyoshi Nojima:** Writing – review & editing. **Hiromichi Naito:** Writing – review & editing, Supervision, Methodology, Conceptualization. **Atsunori Nakao:** Writing – review & editing, Supervision, Methodology, Investigation, Funding acquisition, Conceptualization. **Tetsuya Yumoto:** Writing – review & editing, Supervision, Methodology, Conceptualization.

## Funding

This work was supported by a grant from the Okayama Prefecture Regional Medical and Long-Term Care Comprehensive Securing Fund, administered by the Okayama Medical Association, Japan. The funder had no role in the study design, data collection, analysis, interpretation, manuscript preparation, or the decision to submit the article for publication.

## Declaration of competing interest

The authors declare that they have no competing interests.
