# Supplementary material for: Implementation gap in prehospital termination-of-resuscitation policies for out-of-hospital cardiac arrest with do-not-attempt-cardiopulmonary-resuscitation wishes in Japan: a nationwide survey
Source: Resusc Plus. 2026 Sep 1;32:101474. doi: 10.1016/j.resplu.2026.101474 (PMC13602328; doi:10.1016/j.resplu.2026.101474)
Supplement: Supplementary file 1 [file mmc1.docx]

**Appendix A. Survey Instrument for Medical Control Organizations:**

Prehospital Termination-of-Resuscitation Policies for Out-of-Hospital Cardiac Arrest With Do-Not-Attempt-Resuscitation Wishes in Japan

This supplementary appendix presents the English version of the questionnaire administered to medical control organizations in Japan. The survey was designed to assess policy availability, policy content, and implementation readiness for prehospital termination of resuscitation in out-of-hospital cardiac arrest with do-not-attempt-resuscitation wishes.

In Japan, the medical control system includes prefecture-level medical control councils and regional medical control councils. Please answer at the level of your organization.

Unless otherwise specified, questions refer to the policy status at the time of the survey.

Definitions used in this questionnaire

- OHCA: out-of-hospital cardiac arrest.

- DNAR: a previously expressed wish not to receive cardiopulmonary resuscitation in the event of cardiac arrest.

- TOR: termination-of-resuscitation

- Prehospital TOR for OHCA with DNAR wishes: a local policy that allows EMS personnel to terminate resuscitation in the prehospital setting after the patient’s DNAR wishes have been confirmed, for example through written documentation and/or confirmation with the primary physician or an online medical control physician.

- EMS: emergency medical services.

- MC: medical control.

Participation is voluntary. Responses will be analyzed in aggregate form only.

After reading the explanation above, please indicate your consent to participate.

( ) I have read the explanation and agree to participate.

If your organization has an established policy and related documents that can be shared, please indicate below.

( ) Yes

( ) No

If needed, the study office may contact you separately regarding document sharing.

**Part I. Respondent information**

Please provide information on your organization.

1. Prefecture: (	)

2. Type of responding MC organization:

( ) Prefecture-level MC council

( ) Regional MC council

3. Name of responding MC organization: (	)

**Part II. Policy availability and development status**

Q1. Does your MC organization currently have a prehospital TOR policy for OHCA with DNAR wishes?

Select one answer.

( ) Yes, a policy is in place.

( ) A policy is currently under development.

( ) No policy is in place.

Q2. If you answered “Yes” to Q1, please answer the following.

Q2-1. At what administrative level is the policy discussed and implemented?

Select one answer.

( ) Prefecture-level MC council

( ) Regional MC council

( ) Fire department / EMS agency level

( ) Other: (	)

Q2-2. When did policy implementation begin?

Enter the date.

Year (	)	Month (	)	Day (	)

Q2-3. What is the current revision status of the policy?

Select one answer.

( ) No revision has been made since initial implementation.

( ) The policy has been revised one or more times.

( ) The policy is reviewed and updated regularly.

Q2-4. What were the main triggers for policy development?

Select all that apply.

( ) A case in which the patient or family requested TOR on scene

( ) A request from an administrative authority

( ) Internal review and recommendation within the MC organization

( ) A request or proposal from the local medical association or a related organization

( ) Other: (	)

Q2-5. Which stakeholders participated in policy development?

Select all that apply.

( ) Fire department / EMS agency

( ) Medical association

( ) Nursing association

( ) Prefectural or municipal government

( ) Long-term care facility representatives

( ) Legal professionals

( ) Police

( ) Media

( ) Other: (	)

Q3. If you answered “No policy is in place” or “A policy is currently under development” in Q1, please answer the following.

What best describes the reason your organization does not yet have a policy, or its current plans?

Select all that apply.

( ) A policy is under consideration and is expected to be developed in the near future.

( ) The perceived need is low because such cases are rare or current practice is considered sufficient.

( ) Consensus building among stakeholders has been difficult.

( ) Collaboration with medical institutions is insufficient.

( ) Administrative approval has not been obtained.

( ) Policy development has been deferred because of legal or ethical uncertainty.

( ) The organization is awaiting guidance from higher authorities, such as national or prefectural guidance.

( ) Other: (	)

**Part III. Policy content**

Please answer this section only if your organization reported that a policy is in place.

Q1. Do EMS crews confirm whether a patient with OHCA has DNAR wishes?

Select one answer.

( ) Yes, routinely in all cases.

( ) Yes, but only under specific conditions.

( ) No, not routinely.

Q2. If you answered “Yes, but only under specific conditions” to Q1, in which situations do EMS crews confirm DNAR wishes?

Select all that apply.

( ) Older age, such as an age threshold of 75 years or older

( ) End-of-life status, such as terminal malignancy

( ) Baseline functional dependence, such as total dependence in activities of daily living

( ) A low likelihood of successful resuscitation, such as prolonged downtime or initial asystole

( ) Other: (_________________________________________)

Q3. What are the exclusion criteria for this policy pathway?

Select all that apply.

( ) None

( ) Suspected external causes, such as trauma, self-harm, violence, drowning, or asphyxia

( ) Strong family request for resuscitation

( ) Pediatric patients aged 18 years or younger

( ) Other: (_________________________________________)

Q4. How are DNAR wishes confirmed, and on what basis may resuscitation be terminated?

Q4-1. Can resuscitation be terminated solely on the basis of a family member’s verbal request, without confirmation from the primary physician or an online MC physician?

Select one answer.

( ) Yes

( ) No

Q4-2. Which single verification method is sufficient to permit termination of resuscitation?

Select all that apply, if applicable.

( ) a. Written documentation

( ) b. Real-time telephone confirmation with the primary physician

( ) c. Confirmation by an online MC physician

( ) d. A regional DNAR registry

( ) e. A DNAR card, wristband, or similar identifier

( ) f. Other: (_________________________________________)

( ) None

Q4-3. Which combinations of verification methods permit TOR?

Using the options listed in Q4-2 (a through f), select two items to

indicate each permitted combination. More than one combination may be listed.

(	) + (	)	/	(	) + (	)

Q4-4. If other methods permitting termination of resuscitation are specified in the policy, please describe them.

(_________________________________________)

Q5. Please answer the following questions regarding written documentation used as evidence of DNAR wishes.

Q5-1. Is a locally standardized form available?

Select one answer.

( ) Yes

( ) No

Q5-2. What type of documentation is accepted?

Select one answer.

( ) Only the locally standardized form is accepted.

( ) No standardized form exists; documentation is accepted only under specified conditions.

( ) Any written documentation is accepted regardless of format.

( ) This is not specified in the policy.

Q5-3. If you answered “No standardized form exists; documentation is accepted only under specified conditions,” which documents are accepted?

Select all that apply.

( ) A document signed by both the physician and the patient

( ) A document signed by the patient

( ) Other: (_________________________________________)

Q6. When DNAR wishes are reported by a family member or a similar surrogate, how does the policy address confirmation with the patient’s primary physician?

Q6-1. Does the policy define specific confirmation items that EMS crews should ask the primary physician?

Select one answer.

( ) Yes

( ) No

Q6-2. If you answered “Yes,” what items are confirmed?

Select all that apply.

( ) The patient is in the end stage of life

( ) The patient had expressed a wish not to receive CPR

( ) The cause of arrest is consistent with the primary physician’s prior expectation

( ) Other: (_________________________________________)

Q6-3. If the primary physician cannot be reached, how does the EMS crew proceed?

Select all that apply.

( ) Contact an online MC physician for instructions

( ) Continue CPR in principle and transport the patient to a medical facility

( ) The EMS crew may terminate resuscitation at its own discretion

( ) The EMS crew does not attempt to contact the primary physician

( ) Other: (	)

Q7. Who is authorized to decide on termination of resuscitation?

Select all that apply.

( ) Primary physician

( ) Online MC physician

( ) EMS team leader

( ) Receiving physician other than the primary physician

( ) Other: (	)

Q8. After prehospital termination of resuscitation, which dispositions are permitted under the policy?

Select all that apply.

( ) Handover to the primary physician

( ) Handover to the family

( ) Transport to a medical facility without resuming CPR

( ) Handover to the police

( ) Other: (_________________________________________)

Q9. If the patient is handed over directly to the primary physician, is a time limit specified?

Select one answer.

( ) No

( ) Yes. Maximum waiting time: (	) minutes

Q10. If you answered “Yes” to Q9, please state the rationale for that time limit.

(	)

Q11. If the patient is handed over to the family, is a time limit specified?

Select one answer.

( ) No

( ) Yes. Handover is permitted if the primary physician can make a home visit within (	) hours.

Q12. If you answered “Yes” to Q11, please state the rationale for that time limit.

(	)

Q13. If the patient is handed over to the family after termination of resuscitation, is written family consent required?

Select one answer.

( ) No

( ) Yes

Q14. If handover to the primary physician or family is not possible, how is the case managed?

Select one answer.

( ) Transport to a medical facility with CPR

( ) Transport to a medical facility without resuming CPR

( ) Request police involvement

( ) Not specified in the policy

( ) Other: (	)

**Part IV. Education, dissemination, and public outreach**

Please answer this section only if your organization reported that a policy is in place.

Q1. How is the policy communicated to EMS personnel?

Select all that apply.

( ) Initial training was provided when the policy was introduced

( ) Regular training, such as annual education or simulation, is provided

( ) Manuals or written materials are distributed for self-learning

( ) No specific education or dissemination has been implemented

( ) Other: (_________________________________________)

Q2. How is the policy disseminated to regional medical stakeholders?

Select all that apply.

( ) Briefings or workshops were held with the medical association, hospitals, or both

( ) Guidance documents or leaflets were distributed to medical institutions

( ) The policy was introduced individually to physicians who may serve as primary physicians

( ) No specific dissemination or training has been implemented for medical stakeholders

( ) Other: (_________________________________________)

Q3. Are there any public awareness activities related to DNAR for citizens or patients’ families?

Select one answer.

( ) Yes. Specific materials or opportunities are provided, such as leaflets, public lectures, or website information

( ) Partially. DNAR is addressed within broader initiatives, such as advance care planning activities

( ) No. No specific public awareness activity has been implemented

Q4. Is policy development or dissemination linked to local advance care planning initiatives?

Select one answer.

( ) Strongly linked

( ) Partially linked

( ) Not linked

**Part V. Legal considerations**

Please answer this section only if your organization reported that a policy is in place.

Q1. To what extent has the legal basis for following DNAR wishes in the prehospital setting been clarified within your organization?

Select one answer.

( ) Clearly clarified, including organizational review and shared interpretation

( ) Generally reviewed, but not formally documented

( ) Unclear, and concerns remain

( ) Not reviewed

Q2. How does your organization collaborate with legal stakeholders, such as police or lawyers, in relation to policy operation?

Select one answer.

( ) Active collaboration, including participation in policy development or review

( ) Consultation as needed on a case-by-case basis

( ) No specific collaboration framework
