# Supplementary material for: Implementation gap in prehospital termination-of-resuscitation policies for out-of-hospital cardiac arrest with do-not-attempt-cardiopulmonary-resuscitation wishes in Japan: a nationwide survey
Source: Resusc Plus. 2026 Sep 1;32:101474. doi: 10.1016/j.resplu.2026.101474 (PMC13602328; doi:10.1016/j.resplu.2026.101474)
Supplement: Supplementary file 2 [file mmc2.docx]

**Appendix B. Survey Instrument for Fire Departments:**

2024 Aggregate Experience With Out-of-Hospital Cardiac Arrest With Do-Not-Attempt-Resuscitation Wishes in Japan

This supplementary appendix presents the English version of the questionnaire administered to fire departments in Japan. The survey was designed to collect fire-department-level aggregate data on OHCA cases with DNAR wishes during calendar year 2024 and to evaluate policy application, case disposition, and operational experience.

Please answer at the level of your fire department or EMS agency. This questionnaire asks for aggregated counts for the period from January 1 to December 31, 2024. It does not ask for patient-level data.

Definitions used in this questionnaire

- OHCA: out-of-hospital cardiac arrest.

- DNAR: a previously expressed wish not to receive cardiopulmonary resuscitation in the event of cardiac arrest.

- TOR: termination-of-resuscitation

- Prehospital TOR policy for OHCA with DNAR wishes: a local policy that allows EMS personnel to terminate resuscitation in the prehospital setting after the patient’s DNAR wishes have been confirmed, for example through written documentation and/or confirmation with the primary physician or an online medical control physician.

- Policy application: use of the policy in an OHCA case with DNAR wishes.

- Transport with CPR: transport to a medical facility with ongoing CPR.

- MC: medical control.

- On-scene termination: prehospital TOR performed at the scene under a local policy pathway, without subsequent transport to a medical facility with ongoing CPR.

Participation is voluntary. Responses will be analyzed in aggregate form only.

After reading the explanation above, please indicate your consent to participate.

( ) I have read the explanation and agree to participate.

**Part I. Respondent information**

Please provide information on your organization.

1. Prefecture: (	)

2. Regional MC council: (	)

3. Fire department / EMS agency: (	)

4. Which organization developed the local policy or guidance used in your area for OHCA with DNAR wishes?

Select one answer.

( ) Prefecture-level MC council

( ) Regional MC council

( ) Fire department / EMS agency

( ) Other: (	)

( ) No policy or guidance is available

**Part II. Aggregate 2024 case volume, case disposition, and operational experience**

Please answer this section as far as possible, even if your area does not have a policy.

Q1. Between January 1 and December 31, 2024, how many adult OHCA cases were handled by your organization?

Please exclude cases not transported because of obvious signs of death, interfacility transfers, and cases in which cardiac arrest occurred after EMS contact.

Enter a number.

(	) cases

Q2. For the same period, please provide the following aggregated information for adult OHCA cases with DNAR wishes.

Q2-1. Case profile

Enter numbers or means as requested.

a. Number of OHCA cases with DNAR wishes, excluding cases not transported because of obvious signs of death: (	) cases

b. Mean on-scene activity time for all OHCA cases with DNAR wishes, defined as time from EMS arrival on scene to patient handover: (	) min

c. Mean total mission time for all OHCA cases with DNAR wishes, defined as time from EMS dispatch to return to station: (	) min

d. Number of cases in which DNAR wishes were confirmed by written documentation: (	) cases

e. Number of cases that occurred at home: (	) cases

f. Number of cases that occurred in a long-term care facility: (	) cases

Q2-2. Case disposition for OHCA cases with DNAR wishes

Enter numbers.

a. Number of cases in which prehospital TOR was performed under a local policy: (	) cases

b. Number of cases in which TOR was performed following confirmation with the primary physician: (	) cases

c. Number of cases in which TOR was performed following confirmation with an MC physician: (	) cases

d. Number of cases transported to a medical facility with CPR: (	) cases

Q2-3. Additional information for organizations with a policy in place

Please answer this subsection only if your organization had a policy in place during 2024.

( ) No policy was in place during 2024

a. Number of policy-applied cases that were transported to a medical facility after resuscitation had been terminated on scene: (	) cases

b. Number of policy-applied cases handed over to the primary physician or family after resuscitation had been terminated on scene: (	) cases

c. Mean on-scene activity time for policy-applied cases, defined as time from EMS arrival on scene to patient handover: (	) min

d. Mean total mission time for policy-applied cases, defined as time from EMS dispatch to return to station: (	) min

Q3. During 2024, how many complaints or objections from family members or similar persons were related to these cases?

Enter a number.

(	) cases

Examples include complaints because EMS performed CPR despite the patient’s or family’s wishes, or complaints because EMS did not perform CPR.

Q4. If complaints or objections occurred, how did your organization respond?

Select all that apply.

( ) The issue was resolved through explanation and discussion with the family

( ) The organization consulted higher-level bodies, such as the MC council, medical association, or legal advisors

( ) The policy or operational process was reviewed and revised in response to the case

( ) The issue remained unresolved or was still under discussion

( ) No complaints or objections occurred

( ) Other: (_________________________________________)

Q5. Do EMS personnel report legal concerns when managing OHCA cases with DNAR wishes?

Select one answer.

( ) Frequently

( ) Sometimes

( ) Rarely

( ) Never

Q6. Do EMS personnel report ethical concerns when managing OHCA cases with DNAR wishes?

Select one answer.

( ) Frequently

( ) Sometimes

( ) Rarely

( ) Never

Q7. If legal or ethical concerns are present, how does your organization address them?

Select all that apply.

( ) Provide education or guidance in advance on expected legal issues

( ) Use a consultation system involving online MC physicians, supervisors, or both

( ) Use procedures that confirm and document the patient’s wishes in writing to reduce legal risk

( ) Review cases through an ethics committee or similar process and update operations accordingly

( ) No specific countermeasure is in place

( ) Other: (_________________________________________)

Q8. Does your fire department have a prehospital TOR policy for OHCA with DNAR wishes in place?

( ) Yes

( ) No

**Part III. System readiness and operational challenges in organizations with a policy**

Does your fire department currently have a prehospital TOR policy for out-of-hospital cardiac arrest with do-not-attempt-resuscitation wishes?

( ) Yes

( ) No

Please answer this section only if your area has a policy in place.

Q1. Is there an established framework for information sharing and collaboration with regional medical institutions and long-term care facilities?

Select one answer.

( ) Yes, a formal framework is established

( ) Partially established

( ) Informal only, on a case-by-case basis

( ) None

Q2. Is there an advance information-sharing mechanism for DNAR wishes in patients receiving home care or residing in long-term care facilities?

Select one answer.

( ) Yes, a routine mechanism is available

( ) Partially, but not systematically

( ) No

Q3. Does your organization conduct post-incident review after DNAR-related cases?

Select one answer.

( ) Yes, for all cases or on a regular basis

( ) Yes, as needed for selected cases

( ) No

Q4. Since policy introduction, what operational challenges have actually occurred or been frequently reported?

Select all that apply.

( ) None

On-scene operational issues

( ) There are few eligible cases, which limits opportunities for policy application

( ) Confirmation procedures take time on scene

( ) The policy increases the decision-making and operational burden on EMS personnel

( ) Contacting the primary physician is difficult in some cases

Stakeholder-related problems

( ) Family complaints or objections occur, including requests for transport

( ) Responsibility and inquiries are concentrated on MC physicians

( ) Primary physicians or local medical associations express concern or opposition

System-level challenges

( ) The status of advance care planning in the region is unclear, which complicates decision-making

( ) The legal basis remains uncertain

( ) Regional dissemination is insufficient, and the policy has not been fully adopted
